# Composition and hydrothermal pretreatment and enzymatic saccharification performance of grasses and legumes from a mixed-species prairie

**DOI:** 10.1186/1754-6834-4-52

**Published:** 2011-11-15

**Authors:** Jaclyn D DeMartini, Charles E Wyman

**Affiliations:** 1 Chemical and Environmental Engineering Department, University of California, Riverside, Riverside, CA 92507, USA; 2 Center for Environmental Research and Technology, Bourns College of Engineering, University of California, Riverside, 1084 Columbia Ave, Riverside, CA 92507, USA

## Abstract

**Background:**

Mixtures of prairie species (mixed prairie species; MPS) have been proposed to offer important advantages as a feedstock for sustainable production of fuels and chemicals. Therefore, understanding the performance in hydrothermal pretreatment and enzymatic hydrolysis of select species harvested from a mixed prairie is valuable in selecting these components for such applications. This study examined composition and sugar release from the most abundant components of a plot of MPS: a C3 grass (*Poa pratensis*), a C4 grass (*Schizachyrium scoparium*), and a legume (*Lupinus perennis*). Results from this study provide a platform to evaluate differences between grass and leguminous species, and the factors controlling their recalcitrance to pretreatment and enzymatic hydrolysis.

**Results:**

Significant differences were found between the grass and leguminous species, and between the individual anatomical components that influence the recalcitrance of MPS. We found that both grasses contained higher levels of sugars than did the legume, and also exhibited higher sugar yields as a percentage of the maximum possible from combined pretreatment and enzymatic hydrolysis. Furthermore, particle size, acid-insoluble residue (AcIR), and xylose removal were not found to have a direct significant effect on glucan digestibility for any of the species tested, whereas anatomical composition was a key factor in both grass and legume recalcitrance, with the stems consistently exhibiting higher recalcitrance than the other anatomical fractions.

**Conclusions:**

The prairie species tested in this study responded well to hydrothermal pretreatment and enzymatic saccharification. Information from this study supports recommendations as to which plant types and species are more desirable for biological conversion in a mixture of prairie species, in addition to identifying fractions of the plants that would most benefit from genetic modification or targeted growth.

## Background

The only known resource that can promise to support large-scale, sustainable production of organic chemicals and liquid fuels and reduce dependence on petroleum is lignocellulosic biomass [1-3]. However, owing to the large amounts of biomass and land that would be required to satisfy the world's growing energy demands, there are concerns that biofuels would compete with food for fertile land, and may also threaten biodiversity if natural lands are dedicated to monoculture bioenergy crops [4]. For lignocellulosic biofuels to be produced as sustainably as possible, the ideal feedstock would achieve high biomass yields with minimal or no irrigation and fertilization, be grown on degraded and abandoned agricultural lands, and be converted at high yields to sugars and subsequent fuels and/or chemicals. One such potential feedstock is mixed prairie species (MPS), which has been reported to grow well on agriculturally degraded lands with minimal fertilization, irrigation only during establishment, and low inputs otherwise [4]. Tilman *et al. *[4] found that mixtures exhibiting high levels of biodiversity, in particular those including legumes, also benefit from a self-supply of nitrogen, potentially reducing or eliminating the need for nitrogen fertilizer.

Several studies have looked at cell wall digestibility and sugar release of individual legume or grass species [5-10], but to our knowledge, none has investigated the performance of both grass and legume components that are grown together within a mixed plot. Thus, this study aimed to gain a better understanding of how composition and performance in pretreatment and enzymatic hydrolysis vary for three of the most abundant components within a mixture of prairie species: a C3 grass, a C4 grass, and a legume.

Owing to the strongly heterogeneous nature of the mixture and the individual species themselves, composition and sugar release within each species were examined by fractionating plants and analyzing the resulting anatomical components. To facilitate the analysis, members of each class of plant (a C3 grass (*Poa pratensis; *PP), a C4 grass (*Schizachyrium scoparium; *SS) and a legume (*Lupinus perennis*; LP)) were fractionated into their anatomical components, and the resulting mass fractions analyzed (Table 1). Each anatomical component was then fractionated by particle size, creating a total of 36 samples that were analyzed for chemical composition using a downscaled wet-chemistry method [11]. Each of the 36 samples was then subjected to hydrothermal pretreatment followed by enzymatic hydrolysis in a similar high-throughput, scaled-down system as used for the compositional analysis [12]. With this approach, we evaluated differences in composition and sugar-release performance between both grass and leguminous species, and those between anatomical fractions of each plant. This information allowed us to investigate whether there are factors that control the recalcitrance of both grasses and legumes, which could help in identifying fractions of the plants that would most benefit from genetic modification or targeted growth. Finally, this work might also support recommendations as to which plant types are more desirable for biological conversion in a mixture of prairie species.

**Table 1:** Anatomical composition of tested species^a^

Plant part	Anatomical component mass fraction, %		
	
	*Lupinus perennis*	*Schizachyrium scoparium*	*Poa pratensis*
Stem	37.5	43.1	13.0
Leaf	49.3	18.3	68.3
Petiole	13.3	-	-
Sheath	-	25.2	16.3
Flowers	-	13.1	2.4

^a^Mass fraction of major anatomical components for the three species examined in this study, *L. perennis *(LP), *S. scoparium *(SS), and *P. pratensis *(PP). LP included the stem, petiole and leaf, while SS and PP were divided into the stem, sheath, leaf, and flower. Each of these components was weighed to determine its anatomical mass fraction.

## Results and discussion

### Analysis of grass and legume anatomical fractions

#### Composition of anatomical fractions

**Figure 1:**
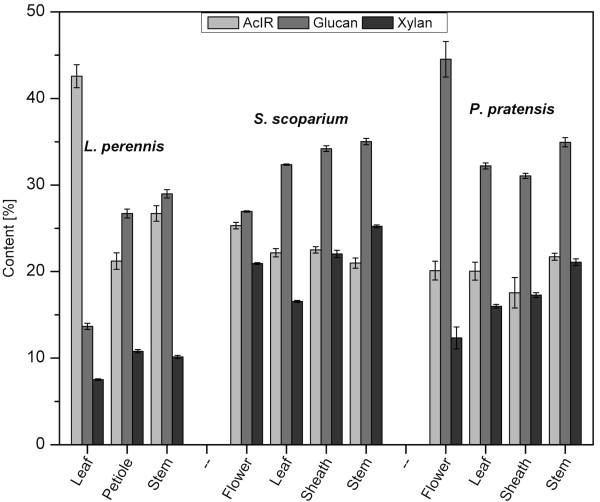
**Composition by anatomy**. Glucan, xylan, and acid-insoluble residue (AcIR) contents as measured by downscaled wet chemistry for each anatomical fraction of the three species tested. Error bars represent the overall standard error from triplicate analyses of each particle-size fraction.

The composition of the anatomical fractions for the three species was compared (Figure 1). The galactan and arabinan contents of the samples are not included in Figure 1 because all of their values were below 3.5%. As shown, the flower fraction from PP had the highest glucan content of any fraction (44.5%), and the stem portion of all species exhibited the next highest: 29.0% for LP, 34.9% for PP, and 35.0% for SS. The stem portion also had the highest xylan content: 10.8% for LP, 21.1% for PP, and 25.2% for SS. With the exception of the leaf fraction from LP, which exhibited the largest AcIR content (42.6%), the AcIR contents of the remaining species and anatomical fractions ranged from 17.5% (PP sheath) to 26.7% (LP stem).

Differing compositions between anatomical fractions have been previously reported for both grasses and legumes. Jung and Vogel [9] showed that the leaves of grasses, including big bluestem (*Andropogon gerardii*) and switchgrass (*Panicum virgatum*), have lower cellulose content than grass stems. Similarly, Aman [13] found that in a legume such as red clover (*Trifolium pratense*), the leaves contained less than half of the sugar content of stems, whereas the lignin content of both legume and grass leaves tended to be lower than that for stems [9,13]. In the present study, although the patterns of sugar concentrations within anatomical fractions agreed with previous studies, the AcIR content, which can serve as an estimate of lignin content, did not entirely coincide. For the two grass species tested, no significant differences were seen between the AcIR content of the stem and leaf fractions. By contrast, the AcIR content of LP leaf was much larger than that of all the other fractions, a particularly unexpected finding, as this fraction contains mostly non-lignified mesophyll cells. The high average AcIR content for LP leaf was mainly associated with two particle-size fractions of this component, the -20/+40 and -40/+60 mesh fractions, which exhibited AcIR contents of 72% and 48%, respectively, compared with values of 17% to 35% for all the other particle-size fractions tested in this study. Because of these unexpectedly high values, these samples were reanalyzed and confirmed to have particularly high AcIR contents. The cause of this behavior is unknown, but it may be attributed in part to the preferential sieving of the anatomical components. Generally, the lignin in legumes is more localized than in grasses [14]. It is possible that the more highly lignified leaf midrib will be more prevalent in the larger particle-size fractions. Alternatively, the high AcIR content in these particular samples may not mean that they have a significantly higher lignin content, but instead may be due in part to an elevated content of acid-insoluble ash and extractives, including proteins and inorganic materials. These components may also explain why the AcIR contents of the grasses were almost identical for both the leaf and stem sections even though the stem portion was expected to have a higher lignin content.

To test whether elevated protein content might have been the cause of the high AcIR values in the LP leaf samples, the nitrogen content of all particle-size fractions of this species was analyzed. The resulting protein values were estimated to be in the range of 7% to 11% for LP leaf depending on particle size, about twice the protein content of 4% to 6% for LP stem. Nonetheless, although protein content might have resulted in a slightly higher AcIR content for some MPS samples, it cannot fully account for the particularly high AcIR content in the LP leaf samples. Thus, it is possible that some other form of unidentified water or ethanol extractives may be responsible for the high AcIR content in legume leaves.

#### Sugar yields of anatomical fractions

**Figure 2:**
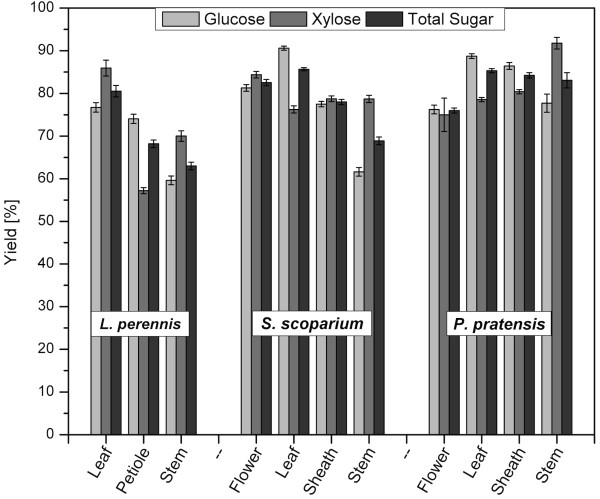
**Sugar yields by anatomy**. Glucose, xylose, and total sugar (glucose + xylose) yields from combined pretreatment and enzymatic hydrolysis for the anatomical fractions of *Lupinus perennis*, *Schizachyrium scoparium*, and *Poa pratensis*. Yields represent the amount of sugar released per amount of sugar available in the biomass (for example, glucose released/glucose in biomass). Error bars represent the standard error of experiments performed with six replicates for each particle-size fraction.

The glucose, xylose, and total sugar (glucose + xylose) yields from combined pretreatment and enzymatic hydrolysis of the anatomical fractions of each species were analyzed (Figure 2). The leaf fraction exhibited the highest glucose yield within each species, ranging from 76.7% in LP to 88.7 and 90.6% in PP and SS, respectively. Conversely, the lowest glucose yields were generally seen in the stem fraction, an observation that was most pronounced in LP and SS, with yields of 59.6 and 61.6%, respectively. The only anatomical fraction that exhibited lower glucose yields than the stem was the flower portion of PP, with a glucose yield of 76.3%, which was slightly lower than that of the same plant's stem (77.7%). Conversely, unlike glucose yields, no clear correlation was found between xylose yield and anatomical fraction. Although xylose yields varied between fractions, ranging from 57.2% in LP petiole to 91.8% in PP stem, no single anatomical fraction exhibited consistently high or low xylose yields for all three species tested.

Other researchers have also reported the influence of anatomical fraction on sugar yields. For example, past work has evaluated sugar yields from husks, leaves, cobs, and stalks of corn (*Zea mays*) in pretreatment and enzymatic hydrolysis [15-17]. Both Montrass and Crofcheck [16] and Garlock *et al. *[17] found the leaves to be less recalcitrant than stalks. Similar work by Anderson *et al. *[18] also found that grass leaves generally exhibited higher *in vitro *dry-matter digestibility compared with stems.

### Analysis of overall species

#### Composition of grass and legume species

**Figure 3:**
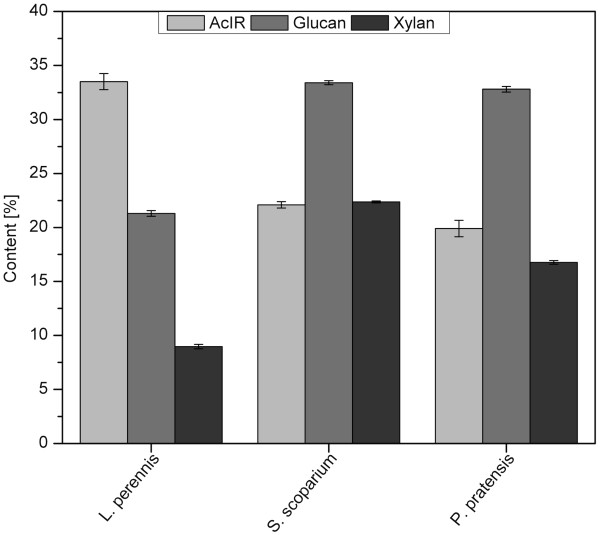
**Composition by species**. Glucan, xylan, and acid-insoluble residue (AcIR) contents as calculated from mass fractions and compositions of anatomical components for *Lupinus perennis*, *Schizachyrium scoparium*, and *Poa pratensis *determined by downscaled wet chemistry. Error bars represent the overall standard error based on the variance of results for anatomical fractions.

Based on the compositions and mass fractions of each of the anatomical parts, the AcIR, glucan, and xylan compositions of the three species were compared. The C4 grass SS contained the highest levels of both glucan (33.4%) and xylan (22.4%), followed by the C3 grass PP with 32.7% glucan and 16.8% xylan (Figure 3). The legume LP contained the lowest levels of sugars, with only 21.3% glucan and 9.2% xylan. By contrast, LP contained 33.5% AcIR, whereas the two grasses had 19.9% (PP) and 22.1% (SS). The lower sugar contents seen for the legume compared with the grasses is not indicative of all species of their kinds. While Torget *et al. *[6] found the legume *Sericea lespedeza *to contain less glucan and xylan than both switchgrass (*Panicum virgatum*) and weeping lovegrass (*Eragrostis curvula*), they later observed that the legume flatpea hay (*Lathyrus sylvestris *L.) had a slightly higher glucan content than reed canary grass (*Phalaris arundinacea*) [7]. Others have shown that whereas glucan content shows no clear trend between grasses and legumes, the xylan content is typically much lower in legumes than in grasses [13]. However, it should also be noted that although some legumes may have a lower content of neutral sugars than do monocot grasses, they probably contain higher amounts of acidic sugars, as do other dicots. As for lignin content, it tends to be significantly higher in legumes than in grasses [6,7,13], consistent with the AcIR results in this study.

#### Sugar yields of grass and legume species

**Figure 4:**
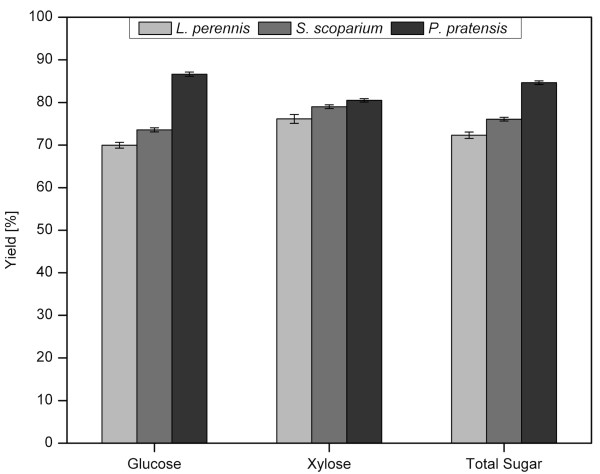
**Sugar yields by species**. Glucose, xylose, and total sugar (glucose + xylose) yields calculated from combined pretreatment and enzymatic hydrolysis of each anatomical fraction and the corresponding mass fractions of each fraction for *Lupinus perennis*, *Schizachyrium scoparium*, and *Poa pratensis*. Yields represent the amount of sugar released per amount of sugar available in the biomass (for example, glucose released/glucose in biomass). Error bars represent the overall standard error based on the variance of results for each anatomical fraction.

The overall sugar yields for the three species were calculated as the percentage of the maximum possible from yields of the individual anatomical fractions and their corresponding mass fractions (Figure 4). The highest overall glucose, xylose, and total sugar (glucose + xylose) yields were for the C3 grass PP, with yields of 86.6%, 80.5%, and 84.6%, respectively. The next highest yields were for the other grass tested, SS, with values of 73.6% glucose, 79.0% xylose, and 76.1% glucose + xylose. Finally, the legume LP responded the most poorly to pretreatment and enzymatic hydrolysis, with glucose, xylose, and glucose + xylose yields of 70.0%, 76.2%, and 72.3%, respectively.

The lower yields from the legume LP are in agreement with past studies that found legume cellulose to be more recalcitrant than that of grasses [5-7,10]. Dien *et al. *[10] attributed this to the differences in plant cell wall structure, and noted that lignin is more uniformly distributed among tissues of grass compared with those of legumes, so that perhaps the lower sugar yields of legumes are associated with those tissues containing higher levels of lignin. Furthermore, in evaluating the differing performance of the two grasses, Galyean and Goetsch [8] reported that cool-season grasses, such as *Poa pratensis*, are more digestible than warm-season grasses, including *Schizachyrium scoparium*, consistent with the results from this study. Those authors attributed this differing behavior to the unique proportions and arrangements of tissues in warm- and cool-season grasses [8]. For example, owing to differences in their photosynthetic pathways and optimal growing temperatures, warm-season grasses have a larger proportion of less digestible stem material [8]. Additionally, the digestibility of the stem and leaf materials themselves differs between warm- and cool-season grasses: digestion of warm-season tissues was reported to be lower, possibly due to a higher concentration of phenolic compounds and a more tightly packed, radial arrangement of tissues [8].

### Evaluating factors that influence glucose yields and glucose release

#### Interpretation of statistical analysis

Although we found that on average, certain anatomical fractions and plant species exhibited greater glucan digestibility, we also evaluated the influence of a larger array of sample characteristics, still including anatomical fraction and species. In particular, analyses of covariance (ANCOVA) and of variance (ANOVA) were performed to investigate, respectively, 1) whether there are individual factors or a combination of factors that influence glucan digestibility and glucose mass release of MPS, and 2) how these factors may be related to each other. To address the first question, the ANCOVA test was performed with three factors (those defined by the experimental setup: plant species, anatomical fraction, and particle size) and four continuous covariates (AcIR content, glucan content, xylan content, and xylose yield). ANCOVA was selected because it takes into account both the effects of individual variables and the interactive effects of multiple variables. When we tested the influence of all of these factors and covariates in a full ANCOVA, we found that only anatomical fraction and AcIR content had a significant effect on glucan digestibility (glucose released/glucose available). To further examine this finding, a reduced model was used, which evaluated the two significant variables (anatomical fraction and AcIR content) identified in the full model. Using this refined model, we found that the anatomical fraction had a highly significant influence on glucan digestibility (*P *< 0.001), whereas the influence of AcIR content was less important (*P *= 0.11). The same analysis was also performed to evaluate the influence of the same set of variables on the glucose mass release (glucose released/total dry biomass). In this case, the full model suggested that anatomical fraction and glucan content had a significant influence. A reduced model confirmed this finding, with both variables having a highly significant influence (*P *< 0.001) on glucose release.

The ANOVA test was performed to evaluate how each of the individual four covariates defined in the previous analyses were related to the three factors (plant species, anatomical fraction, and particle size). Although ANOVA does not consider interactive effects, it is useful in determining the influence of controllable experimental factors on results and in future model development to predict glucan digestibility and glucose mass release with these factors. Thus, glucose yield, glucose mass release, AcIR content, glucan content, xylan content, and xylose yield were defined as the response variables. The significance of the factors' influence on the response variables is summarized in Table 2. As shown by the low *P *values, all of the factors considered influenced most of the response variables. For example, anatomical fraction had an effect on AcIR content (*P *= 0.03) and glucose mass release (*P *= 0.02) and an even stronger influence on glucan content (*P *< 0.001), xylan content (*P *= 0.001), xylose yield (*P *< 0.001), and glucan digestibility (*P *< 0.001). Unlike anatomical fraction, particle size significantly influenced only two variables, the glucan and xylan contents, whereas plant species influenced all variables except glucan digestibility. The results from this test further suggest that the only experimental factor that significantly influenced the glucan digestibility of all samples was the anatomical fraction, whereas both plant species and anatomical fraction influenced glucose mass release. Particle size did not influence either glucan digestibility or glucose mass release.

**Table 2:** Interactive effects between response variables and experimental factors^a^

Response variable	Factor		
	
	Plant species	Anatomical fraction	Particle size
AcIR content	< 0.001	0.03	0.11
Glucan content	< 0.001	< 0.001	0.02
Xylan content	< 0.001	< 0.001	0.01
Xylose yield	0.05	< 0.001	0.80
Glucan digestibility	0.34	< 0.001	0.12
Glucose mass release	< 0.001	0.02	0.45

^a^The significance of interactive effects is represented by the *P*-value. The significance (*P*-value) of the influence of the experimental factors (plant species, anatomical fraction, and particle size) on response variables (AcIR content, glucan content, xylan content, xylose yield, glucan digestibility, and glucose mass release) is displayed. The *P *values are calculated from individual ANOVA tests and are displayed for each interaction.

#### The importance of anatomical composition

Both statistical analyses stressed the importance of anatomical fraction on sample composition, glucan digestibility, and glucose mass release. ANOVA showed that anatomical fraction had a strong effect on glucan, xylan, and AcIR contents, whereas ANCOVA analysis clearly showed that when both individual and interactive effects were considered, anatomical fraction was the variable that most significantly influenced glucan digestibility of the MPS considered here. The strong influence of anatomical fraction is logical because it will define the distribution and relative amounts of various tissue types, which in turn affect cell shape, size, wall thickness, and corresponding cell wall surface area to volume ratio. All of these factors have previously been suggested to affect digestibility [19-22]. In general, the leaf fraction exhibited higher glucose yields; the stem fraction tended to be significantly more recalcitrant to glucose release; and the flower, sheath, and petiole fractions exhibited intermediate performance compared with leaf and stem. These observations can be attributed to differences in tissue type and distribution for the anatomical fractions. Leaves are primarily composed of thin-walled (approximately 0.15 μm thick) and loosely arranged mesophyll cells with a high proportion of intercellular airspace and few wall contacts between cells, rendering them easily disrupted and digested [14,21]. Conversely, stems contain high levels of vascular tissue that are rich in recalcitrant and highly lignified xylem elements [14,15]. Furthermore, stems also contain a higher proportion of thick-walled parenchyma (approximately 1.0 μm) and sclerenchyma (approximately 2.4 μm) cells [21] that are probably more difficult to break down. The general anatomy of sheaths and petioles has been reported to be between that of the leaf blade and stem [14], possibly explaining their intermediate performance.

In contrast to the anatomical fraction, the particle size, AcIR content, xylose yield, or xylan content did not strongly influence glucan digestibility of the MPS samples. Zeng *et al. *[23] also reported that smaller particle sizes did not consistently result in higher glucose yields from hydrothermal pretreatment and enzymatic hydrolysis. Although it might seem intuitive that smaller particle sizes improve enzyme effectiveness because of their higher surface area to volume ratio, it may be that pretreatment disrupts biomass particles and increases accessible surface area and pore volume sufficiently that differences caused by particle size are masked [23,24].

The lack of significant influence of AcIR content on glucose yield was somewhat unexpected, as glucose yield has been commonly reported to be inversely related to lignin content (which is by far the largest component of AcIR). However, as explained above (section on 'Composition of anatomical fractions'), AcIR content only provides an estimate of the Klason lignin amounts because the downscaled analysis procedure measures the total acid-insoluble residue including the acid-insoluble ash. The lack of influence of AcIR content on glucose yield disagrees with many past studies showing that increased lignin content adversely affected glucan digestibility, caused in part by restriction of enzyme access and non-productive binding [25-27]. It is possible that AcIR measurements of grasses and legumes are not a sufficient estimate of their lignin content, and that if glucose yields were evaluated versus the true lignin content, a significant effect might be seen. However, it is still interesting to note that AcIR as a whole, which is comprised mostly of lignin, does not seem to have a significant direct negative effect on yields.

The absence of a significant influence of xylose yield on glucan digestibility was also unexpected, because it has often been reported that glucan digestibility correlates positively with hemicellulose removal [28-30]. Yet, we found no strong correlation to support this hypothesis in the grasses and legumes tested in this study. As an example, for two different samples that both exhibited a xylose yield of 75%, the corresponding glucose yields were 57% and 93%, suggesting that other factors must also contribute significantly to glucan digestibility.

#### Implications for biomass in mixed-species prairies

Good glucose and xylose yields were obtained for the most common grass and legume species that comprised the plot of MPS, especially considering that pretreatments were performed with water alone, at a maximum temperature of 180°C, owing to pressure limitations in the steam chamber. Use of acid or higher temperatures would probably improve yields and might be particularly beneficial for legumes [6,7]. This study also provides insight into possible strategies to improve the conversion characteristics of MPS. The results indicate that plant anatomy is a key factor in grass and legume recalcitrance, and furthermore, that leafy material responds better to hydrothermal pretreatment and enzymatic hydrolysis than do stems, suggesting that genetic modification of stems could be most productive. Also along these lines, methods to increase the leaf:stem ratio would improve overall glucose yields from combined pretreatment and enzymatic hydrolysis. However, despite the differences in glucan digestibility between the various anatomical fractions, the mass of sugar produced per total mass of biomass must also be considered. As such, in this study, the stem released more sugar in LP and PP (280 and 500 g glucose + xylose/kg dry biomass, respectively) than did the leaves (200 and 430 g glucose + xylose/kg dry biomass, respectively), whereas in SS, the amounts were identical for both (440 g glucose/kg dry biomass). This consideration diminishes the consequences of lower sugar yields from stems, and suggests that breeding targets for improved conversion should be directed at stems in order to capitalize on their higher sugar contents.

A similar analysis was used for the three species tested. Although PP exhibited higher glucose yields than SS, the mass of sugar produced per total feedstock mass was almost identical for the two grasses. By contrast, the legume LP released almost half as much glucose + xylose as the grasses. Nevertheless, despite the low sugar yields and mass release exhibited by LP, legumes constitute an integral part of MPS because of their ability to fix nitrogen and thereby reduce or eliminate the need for nitrogen fertilizer. Hence, one potential solution could be to plant other legumes that offer similar nitrogen-fixing benefits but can produce higher structural carbohydrate contents and sugar yields than LP. Alternatively, it may be advantageous to select a harvest time that maximizes the grass:legume ratio so that the agricultural benefits of legumes can still be obtained while reducing their negative effect on the biological conversion yields. For cool-season legumes such as LP, this is a very real possibility. In the upper Midwest of the USA, in which the plants for this study were grown, most of the legumes have dried and fallen to the soil surface, releasing nitrogen, by mid-July, whereas the warm-season grasses are near peak growth at this time. Thus, if harvest time occurs during the period at which the grasses begin to senesce, such as late September in the upper Midwest USA, the grass:legume ratio will strongly favor grasses. Additionally, nitrogen loss will be reduced if a harvest time that occurs after the senescence of warm-season grasses is chosen.

## Conclusions

Analysis of the most abundant legume and C3/C4 grass species within a mixture of prairie species showed that the grasses contained higher levels of sugars compared with the legume. The grasses also exhibited higher sugar yields from combined hydrothermal pretreatment and enzymatic hydrolysis, demonstrating that they are the more desirable components in the MPS for conversion to sugars and subsequent fuels and chemicals. In analyzing the influence of a variety of sample characteristics on the recalcitrance of grass and legume MPS, we found no evidence to suggest a direct significant effect of particle size, plant composition, or xylose yield. Instead, plant anatomy was found to be the most influential factor for both glucan digestibility and glucose mass release, suggesting that breeding and harvest methods to control anatomical composition might be an important route to improving sugar yields from MPS.

## Methods

### Biomass samples

A plot of MPS containing 16 different species was planted in the spring of 1994 in Cedar Creek Ecosystem Science Reserve, Minnesota, MN, USA. The species in this plot were grown on nutrient-poor sandy soils, burned annually in the spring, and grown without irrigation or fertilization before the samples were obtained in 2008 by the University of Minnesota. The plot produced 3.38 tons/hectare of fully dried above-ground harvested biomass, and samples for this study were collected from a section 6 × 0.1 m wide. A portion of the plot was collected and sent unsorted to the University of California Riverside (UCR) to represent the entire mixture, while another portion was sorted and sent to UCR as individual species, from which their weight and corresponding percentage of the plot that they comprised was determined. Table 3 shows the 12 species that were identified in the plot at harvest in 2008, with the percentage weight of the plot that each represents. It is important to note that the biomass fractions shown are from a sampling performed in early July, whereas the biomass tested in this study is from late autumn when the C4 grasses, including *Andropogon gerardi*, *Sorghastrum nutans*, and *Schizachyrium scoparium*, would probably have outgrown the others, and therefore would make up a larger fraction of the biomass.

**Table 3:** Plant species comprising the plot^a ^of mixed prairie species

Species name	Classification	Mass fraction, %	Cumulative mass fraction^b^, %
*Schizachyrium scoparium*	C4 grass	31.5	31.5
*Lupinus perennis*	Legume	27.3	58.8
*Andropogon gerardii*	C4 grass	14.7	73.5
*Poa pratensis*	C3 grass	12.1	85.6
*Lespedeza capitata*	Legume	8.4	94.0
*Monarda fistulosa*	Non-leguminous forb	3.7	97.7
*Sorghastrum nutans*	C4 grass	1.3	99.0
*Asclepias tuberosa*	Non-leguminous forb	0.2	99.2
*Achillea millefolium*	Non-leguminous forb	0.1	99.3
*Agropyron repens*	C3 grass	0.1	99.4
Miscellaneous litter	-	0.8	100.2

^a^The plot from which these species were obtained produced 3.38 tons/hectare of fully dried aboveground harvested biomass.

^b^Cumulative mass fraction does not add up to 100.0% because of rounding.

### Material preparation

From the 12 sorted species, the most abundant C3 grass (PP), C4 grass (SS), and legume (LP) were selected for further analysis. Each of these air-dried materials (moisture content approximately 7%) was first divided into its anatomical components. For the legume, this comprised the stem, petiole, and leaf, whereas for the grasses, the materials were divided into stem, sheath, leaf, and flower. Each of these fractions was weighed to determine the anatomical mass fraction for each species (Table 1).

After fractionation into anatomical components, each of the subsamples was then milled (Wiley Laboratory Mill Model 4; Arthur H. Thomas Company, Philadelphia, PA, USA) until it passed through a 20-mesh screen (< 0.85 mm). Material was collected and then sieved using USA Standard Testing Sieves (Fisher Scientific Company, Pittsburg, PA, USA), from which different particle-size fractions were collected: -20/+40 mesh (425 <*x *< 850 μm), -40/+60 mesh (250 <*x *< 425 μm), -60/+80 mesh (180 <*x *< 250 μm), and < 80 mesh (*x *< 180 μm). All subsequent experiments were performed on the individual particle-size fractions obtained from the anatomical components of each species. It should be noted that for PP, there was not enough flower, sheath, or stem material to produce all four particle-size fractions. As a result, the PP flower sample included all material that fell through the 20-mesh screen, whereas the PP sheath and stem samples were collected in two fractions: -20/+60 and < 60 mesh.

### Compositional analysis

Glucan, xylan, arabinan, galactan, mannan and AcIR contents were determined by performing a downscaled wet-chemistry compositional analysis coupled with high-performance liquid chromatography (HPLC) and gravimetric methods to allow analysis of the small amounts of materials available [11]. This procedure is nearly identical to conventional procedures [31], and produces virtually identical results; however, it uses 100 times less biomass (3 mg versus 300 mg) and can be automated using a solid- and liquid-dispensing robotics platform (Core Module Standard Configuration 2 equipped with Sartorius WZA65-CW balance and 10biomass-dispensing hoppers of 25 mL capacity; Freeslate, Inc., Sunnyvale, CA, USA). After two-stage acid hydrolysis in the downscaled system, the neutralized sugar hydrolyzates were measured by HPLC, while the AcIR contents were determined by gravimetric methods to provide an estimate of Klason lignin content. Unlike the conventional method, this downscaled procedure measures the total AcIR including the acid-insoluble ash, and as a result, cannot measure lignin content directly. Additionally, the composition of the unsorted material was determined by the conventional scaled-up procedure described by Sluiter *et al. *[31].

To test for the protein content in the LP leaf samples, the nitrogen content was measured (EATM 112 N/Protein plus CHNS/O Analyzer; CE Elantech, Lakewood, NJ, USA) with L-aspartic acid (10.52% N, 36.09% C) as a standard. A nitrogen factor of 6.25 was used to estimate the resulting protein content of the samples [32].

### Pretreatment and enzymatic hydrolysis

All samples were subjected to hydrothermal pretreatment followed by enzymatic hydrolysis to determine total glucose and xylose released from the combined operations using a high-throughput pretreatment and enzymatic hydrolysis (HTPH) system described in detail previously [12]. In this study, 4.5 mg of dry biomass were loaded into individual wells of a custom-built metal well plate using a robotics platform (Core Module; Freeslate, Inc.). The well plate used in this study differs from that described by Studer *et al. *[12] in that the wells are larger, employing a reaction mass of 450 mg as opposed to 250 mg. In addition, the individual wells themselves are free-standing on a brass plate, as opposed to being press-fit into an aluminum plate as per the previous report, enabling the robot's four-pronged gripper to pick up and move individual wells to the balance for accurate weighing and biomass dispensing. After the well plate was loaded with biomass samples, it was taken off the robot's deck, and 445.2 μl of deionizedwater was transferred into all wells using an eight-channel pipettor (30-300 μl; Eppendorf, Hamburg, Germany) to achieve a solids loading of 1% w/w. A flat silicone gasket (thickness 1.5875 mm, durometer hardness A40) was laid on top of the open ends of the wells with a thin stainless steel sheet (0.1016 mm) then placed on top of the gasket. This entire assembly was then clamped between two stainless steel plates using spring washers (flat load 1,500 N) and wing nuts. Next, the sealed plate assembly was placed in a custom-built steam chamber for pretreatment with condensing steam [12] provided by a Fulton steam boiler (FB-075-L, Fulton Companies, Pulaski, NY, USA).

After a pretreatment of 44 minutes, the reaction was quenched by opening the valve to the chamber to release the steam, followed by flooding the chamber with cold water. Afterwards, the plate assembly was removed and opened, and 33.7 μl of a mixture of 1 mol/l citric acid buffer (pH 4.95), sodium azide solution (1 g/L), and enzyme was added to the pretreated biomass slurry in each well using an eight-channel pipettor (10-100 μl, Eppendorf). The resulting mixture contained 6.750 mL of buffer, 1.350 mL of sodium azide solution, and 2.002 mL of a dilute cellulase (Spezyme CP, lot no: 3016295230, 116 mg protein/mL) and xylanase (Multifect, lot no: 301-04021-015, 56.6 mg protein/mL) (both Genencor, Palo Alto, CA, USA) solution prepared at a protein mass ratio of 3:1, respectively, to which deionized water was added at a volume ratio of 3:1. The resulting enzyme loading corresponded to 75 mg of cellulase + 25 mg xylanase per gram of glucan + xylan in the raw biomass for the unsorted material, which had a composition of 26.7% glucan, 12.5% xylan, and 20.3% Klason lignin, as determined by the conventional analytical procedure. After addition of the enzyme/buffer/biocide solution, the entire plate assembly with silicone gasket was re-assembled and placed on its side in an incubation shaker (Multitron Infors-HT, ATR Biotech, Laurel, MD, USA) set at 50°C and 150 rpm.

After 72 hours, the well plate was removed from the shaker, and the slurry from each individual well was transferred to 2.0 mL polypropylene (PP) centrifuge tubes (Safe-Lock 2.0 mL test tubes, Eppendorf). After centrifugation (5415 D; Eppendorf) at 18,200 g for 5 minutes, 300 μL of hydrolyzate were transferred to HPLC vials for analysis.

Before running all subsamples in the HTPH system, the unsorted MPS was used to establish a pretreatment yield curve from which a condition could be chosen for subsequent testing. The -20/+40 and -40/+60 mesh fractions of the unsorted MPS were used in the optimization, which was performed at five different hydrothermal pretreatment times, all at a temperature of 180°C. Additionally, the two size fractions of the unsorted MPS were subjected directly to enzymatic hydrolysis without any prior pretreatment. Based on these results, a suboptimal pretreatment severity of 4.0, corresponding to 44 minutes at 180°C, was selected to reduce xylose degradation but still achieve reasonably high sugar yields.

### Sugar analysis

Sugar concentrations for compositional analysis were measured by HPLC (Alliance 2695 equipped with 2414 RI detector; Waters, Milford, MA, USA) with an Aminex HPX-87P column (BioRad, Hercules, CA, USA) heated to 85°C using distilled and deionized water as the eluent, while sugar concentrations from HTPH testing were measured on an Aminex HPX-87H column (BioRad, Hercules, CA, USA) heated to 65°C using the same HPLC configuration but with 0.005 mol/L sulfuric acid as the eluent. Both measurements used an eluent flow rate of 0.6 mL/min.

### Statistical analysis

As mentioned above, each anatomical fraction was further divided into subsamples based on particle size (see 'Material preparation'), for which compositional analyses were performed in triplicate, and HTPH sugar release was measured in six replicates. To determine the composition and sugar release of an entire anatomical fraction, the following equation was used to combine results for the subsamples sorted by particle size:

(1) $$X=\Sigma {\text{m}}_{i}\;{\bar{\text{x}}}_{\text{i}},$$

where *X *is the desired composite result for the entire anatomical fraction (such as glucan content or glucose yield), m_*i *_is the mass fraction of sub-sample *i *with a particular particle-size range, and ${\bar{x}}_{i}$ is the corresponding average result (such as glucan content or glucose release) for sub-sample *i *for the set of replicates analyzed. To enable calculation of the overall standard error for an entire anatomical fraction, the variance was computed and summed over each subset, as shown below:

(2) $$\text{Y}=\sqrt{\Sigma m_{i}{}^{2}[variance{\left(\bar{x}\right]}_{i})}=\sqrt{\Sigma {\text{m}}_{i}{}^{2}\left(\frac{s_{i}{}^{2}}{n}\right)}$$

where Y is the standard error of a result (such as glucan content or glucose yield) for an entire anatomical fraction, m_*i *_and ${\bar{x}}_{i}$ are as defined above, and the variance of ${\bar{x}}_{i}$ is calculated by dividing the square of its standard deviation *s_i _*by the number of replicates (n). The same statistical approach was applied to analyze compositions, sugar release, and corresponding standard errors, for an entire species based on the results for that species' anatomical fractions. Error bars on plots represent the overall standard error as described above.

All statistical analysis was performed using the SAS software package (version 9.2; SAS Institute, Cary, NJ, USA). To evaluate the influence of individual or combinations of sample characteristics on glucan digestibility and glucose mass release, ANCOVA was performed. The three factors were those defined by the experimental setup, including plant species, anatomical fraction, and particle-size fraction. The four continuous covariates were AcIR content, glucan content, xylan content, and xylose yield. To further evaluate how the individual four covariates, as well as the glucan digestibility and glucose mass release, were related to the three factors (plant species, anatomical fraction, and particle size), we used ANOVA. For both analyses, all samples were evaluated including the individual particle sizes of the separate anatomical fractions for all 3 species (36 samples in total).

## List of abbreviations used

AcIR: acid-insoluble residue; HPLC: high-performance liquid chromatography; HTPH: high-throughput pretreatment and enzymatic hydrolysis; LP: *Lupinus perennis; *MPS: mixed prairie species; PP: *Poa pratensis; *SS: *Schizachyrium scoparium*.

## Competing interests

CEW is cofounder of Mascoma Corporation and chair of their Scientific Advisory Board. CEW is also member of the Scientific Advisory Board of Mendel Biotechnology, Inc. CEW is also founding Editor in Chief of this Journal BfB.

## Authors' contributions

JDD designed and performed the research, analyzed the data and wrote the paper. CEW coordinated the research and helped to finalize the manuscript. All authors read and approved the final manuscript.
